# Factors Influencing the Intention of Actors in Hospitals to Use Indoor Positioning Systems: Reasoned Action Approach

**DOI:** 10.2196/28193

**Published:** 2021-10-05

**Authors:** Johannes Wichmann, Michael Leyer

**Affiliations:** 1 Chair of Service Operations Institute of Business Administration Rostock University Rostock Germany; 2 Wismar Business School Wismar University Wismar Germany; 3 Department of Management Queensland University of Technology Brisbane Australia

**Keywords:** indoor positioning systems, indoor navigation, indoor localization, hospital, clinic, reasoned action approach, survey, hospital visitors, hospital employees

## Abstract

**Background:**

Indoor positioning systems (IPS) have become increasingly important for several branches of the economy (eg, in shopping malls) but are relatively new to hospitals and underinvestigated in that context. This research analyzes the intention of actors within a hospital to use an IPS to address this gap.

**Objective:**

To investigate the intentions of hospital visitors and employees (as the main actors in a hospital) to use an IPS in a hospital.

**Methods:**

The reasoned action approach was used, according to which the behavior of an individual is caused by behavioral intentions that are affected by (1) a persuasion that represents the individual’s attitude toward the behavior, (2) perceived norms that describe the influence of other individuals, and (3) perceived norms that reflect the possibility of the individual influencing the behavior.

**Results:**

The survey responses of 323 hospital visitors and 304 hospital employees were examined separately using SmartPLS 3.3.3. Bootstrapping procedures with 5000 subsamples were used to test the models (one-tailed test with a significance level of .05). The results show that attitude (*β*=.536; *P*<.001; *f²*=.381) and perceived norms (*β*=.236; *P*<.001; *f²*=.087) are predictors of hospital visitors’ intention to use an IPS. In addition, attitude (*β*=.283; *P*<.001; *f²*=.114), perceived norms (*β*=.301; *P*<.001; *f²*=.126), and perceived behavioral control (*β*=.178; *P*=.005; *f²*=.062) are predictors of hospital employees’ intention to use an IPS.

**Conclusions:**

This study has two major implications: (1) our extended reasoned action approach model, which takes into account spatial abilities and personal innovativeness, is appropriate for determining hospital visitors’ and employees’ intention to use an IPS; and (2) hospitals should invest in implementing IPS with a focus on (a) navigational services for hospital visitors and (b) asset tracking for hospital employees.

## Introduction

### Overview

Hospitals are characterized by high levels of physical movement, with a constant stream of temporary visitors (patients and related visitors), personnel, and mobile technical equipment operating in different locations. While efficiency is a concern, it is also of the utmost importance to ensure high levels of hygiene to avoid contamination and the spread of disease, a necessity highlighted by the COVID-19 pandemic. Consequently, preventing the spread of disease by improving hygiene [1] has been the subject of numerous studies [2,3]. Indoor positioning systems (IPS) can support hospitals’ efforts to improve hygiene for visitors and employees in three main ways. First, IPS in hospitals can facilitate wayfinding [4] and support measures against hospital-related infections, such as social distancing [5,6]. Second, IPS can help employees find hospital assets [7] and enable patients to move through different departments [8]. Third, IPS can be used to monitor patients in need of assistance (eg, those with dementia) [9].

Until now, the market penetration for IPS in hospitals has been low because of high implementation costs—roughly US $10200 for approximately 9290 m² [10]. However, as radio-frequency identification tags and Bluetooth beacons have become cheaper, implementing IPS in hospitals is more attractive for hospital management [11]. Commercial implementations of IPS in hospitals in Germany [12] and the United States [13] provide examples of growing interest. Nonetheless, when assessing the costs and benefits, it is important to consider potential user acceptance issues, as high usage rates are necessary to obtain the full benefits of IPS.

Research on the adoption of health care tracking apps has shown the importance of acceptance, notably in the context of COVID-19 [14]. The results highlight the importance of functional and trust-related factors in the use of and intention to use such apps [15]. Some studies have applied model-driven approaches, such as the technology acceptance model [16,17], to different IPS contexts [18]. However, in the hospital context, the only relevant study is that of Anagnostopoulos et al [19], who investigated the IPS needs of employees at Geneva University Hospital.

To investigate the intention of actors in hospitals to use IPS, we adopted the well-established reasoned action approach (RAA) as a causal model to identify relevant influencing factors. The RAA identifies reasons for a specific behavior by considering behavioral, normative, and control beliefs [20]. We surveyed 323 hospital visitors and 304 hospital employees in Germany. We set up a structural equation model (SEM) for both groups that includes factors relevant to the intention to use an IPS.

Our results contribute to understanding which factors influence the intention of actors (ie, hospital visitors and employees) to use systems or applications (ie, IPS) in the health care management context. We show that the RAA, extended to include spatial abilities, can explain the intentions of two major stakeholder groups to use systems in the context of health care management. Hospitals wishing to improve hygiene can apply these insights to encourage IPS usage. This will help tackle a range of issues, from the threat of multiresistant germs to restrictions on hospital visitor numbers during a pandemic. Therefore, we recommend that hospitals invest in the implementation of IPS, taking stakeholder-specific requirements into account.

This article is organized as follows: the second and third sections clarify the theoretical background to the research and introduce the hypotheses and research model. The fourth section describes the materials and methods, and the fifth section presents the results, which are discussed in the sixth section. The final section concludes the research, clarifies its implications, and provides an outlook for further investigations.

### Theoretical Background

#### Indoor Navigation/Indoor Localization

**Figure 1:**
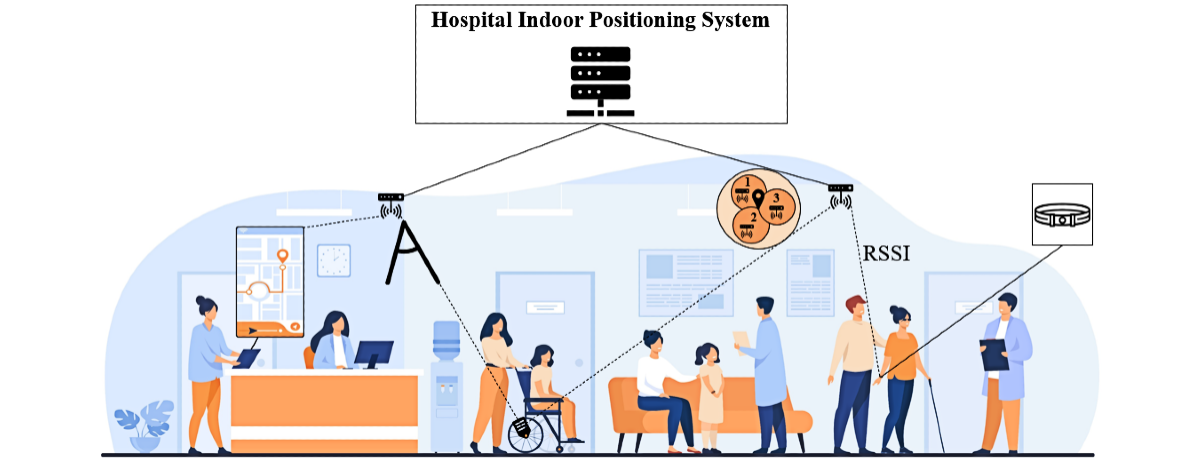
Functional setup of an exemplary positioning system in a hospital [25–28]. RSSI: received signal strength indication.

An IPS determines the specific position of an individual or an asset [21] using an algorithm that estimates the position of a mobile client. Figure 1 shows how such connections can be established in a hospital setting [22] using a mobile device [23], a tag (ie, attached to a wheelchair), and a wristband [24]. These devices are connected by a set of reference points (ie, routers [25]) within a predefined area [26]. This allows different localization techniques such as Bluetooth or Wi-Fi to be combined with calculation principles to determine specific positions. Frequently used calculation principles are triangulation (represented here by the three circles) and trilateration (represented by the triangle), which use the received signal strength indication of the relevant localization technique [27]. An IPS of this type can be used to track patients in urgent care [8] or to locate insulin pumps [28], ultimately reducing waiting times and redundant activities.

#### Research on Indoor Navigation/Indoor Localization in Hospitals

Navigation applications allow the tracking of individuals by connecting localization data with personal data [29]. Research on health care tracking apps has shown the importance of social [14] and behavioral factors [14,15] in relation to usage rates and intention to use. For example, research on COVID-19 apps has established that trust and privacy [15,30,31], as well as voluntary and temporary use, are important factors in acceptance [31]. In addition, a lifestyle that prioritizes hygiene has been identified as a major predictor of using a COVID-19 app, although evidence from Singapore suggests that demographics and situational characteristics are less relevant [32]. Although this previous research has identified factors that may be relevant, it focuses on general app usage. Therefore, we extend it by introducing a specific spatial and organizational context, namely the use of IPS in hospitals.

Model-driven approaches have been adopted in IPS research to account for the navigational requirements of users [18,33]. For example, Arning et al [18] applied the technology acceptance model [16,17] to an IPS that operates using a screen (eg, a smartphone) and a pico-projector. They found strong evidence that disorientation is the most important predictor of screen and projector acceptance. However, their research was limited to young people (ie, university students between the ages of 21-28 years) and may not be generalizable to other age groups. It should also be noted that the technology acceptance model does not include social influences, which are likely to be an important predictor for intention to use an IPS [34].

Within the hospital context, the only relevant study is that of Anagnostopoulos et al [19], who investigated the IPS needs of staff at Geneva University Hospital. They identified five key features of an app: (1) it should show the trajectory toward a destination on a map; (2) it should consider the mobility capabilities of users; (3) it should protect the individual’s privacy; (4) it should estimate the position accurately; and (5) it should not require an internet connection to function properly. However, as these results were obtained from a specific case study, they may not be generalizable to users in other contexts.

#### The Reasoned Action Approach

**Figure 2:**
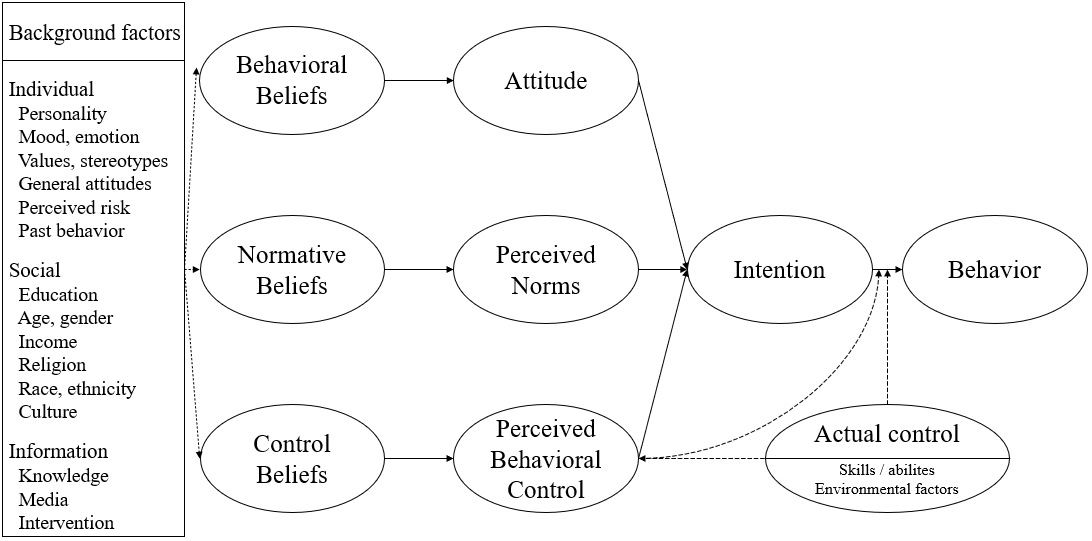
The reasoned action approach (RAA) according to Fishbein et al [20].

The RAA is a well-established psychological approach based on the theory of reasoned action [35-38], which is widely accepted in psychological studies [39] and is appropriate for ascertaining individual behavior. According to the RAA, individual behavior is caused by behavioral intentions that are rooted in (1) a persuasion that influences the individual’s attitude toward the behavior; (2) perceived norms that describe the influence of other individuals; and (3) the opportunity for the individual to affect the behavior, referred to as perceived behavioral control [20]. Figure 2 represents the RAA in greater detail.

An individual's attitude regarding a certain behavior is influenced by his or her beliefs concerning the characteristics and attributes related to the behavior. Thus, an attitude is affected by individual consequences that emerge through assessments of whether or not the behavior is desirable. Therefore, the individual is influenced by whether the behavior is endorsed or opposed by other individuals or groups (those who are most important to her or him in terms of the relevant behavior). The aggregation of motivation and perception assessments for all relevant referent groups is referred to as perceived norms [35,36,38].

Perceived behavioral control determines whether an individual is capable of or directly controls a specific behavior. It is defined by control beliefs that reflect the individual's key personal or situational aspects in relation to the behavior. Ultimately, performing a specific behavior involves the comparison and selection of attitudes, perceived norms, and perceived behavioral controls associated with each of the alternative behaviors in the choice set [40]. Considering these factors together makes it possible to ascertain the likelihood of an individual performing a specific behavior.

### Hypotheses and Research Model

The RAA is a framework that has to be adjusted to a specific context [20]. In this study, we apply it to the hospital context to predict intention to use an IPS.

First, behavioral beliefs are important for ascertaining the value that an individual perceives in using an IPS. These beliefs cover whether an IPS is perceived as helpful in finding the right location or tracking an object. The positive or negative feelings an individual has toward using an IPS in a hospital (the individual’s attitude) are rooted in those beliefs. For the purposes of this study, positive feelings are taken as how the individual feels, as it is the individual who determines whether an IPS is beneficial, satisfactory, relevant, and pleasant to use [41]. The RAA then states that if an individual's attitude toward an IPS is positive, the individual will have a higher intention to use the IPS [20,40]. These considerations lead to the following hypotheses:

- H1: The higher the behavioral beliefs concerning the use of an IPS in a hospital, the more positive an individual's attitude regarding the IPS.
- H2: The more positive an individual's attitude concerning the use of an IPS in a hospital, the higher the intention to use the IPS.

Second, in line with RAA research, we represent the attitudes of other relevant individuals and groups as normative beliefs (subjective norms) [20,42,43]. For hospital visitors, we define family and close friends as relevant social influence groups. For hospital employees, we define immediate colleagues, colleagues in related functional areas, and superiors as relevant influence groups. Normative beliefs generate perceived pressure or motivation, according to whether the individual thinks using an IPS is supported or urged by the reference groups. As implementing an IPS system can be very complex, and the demands on the time and effort of the individual may be high [44], hospital visitors and employees are likely to seek insights from other individuals and groups. In terms of the RAA, the more positive the perception of support from the reference groups, the higher the intention to use an IPS in a hospital. These considerations lead to the following hypotheses:

- H3: The higher the normative beliefs concerning the use of an IPS in a hospital, the more positive an individual’s perceived norms regarding the IPS.
- H4: The more positive an individual’s perceived norms regarding the use of an IPS in a hospital, the higher the intention to use the IPS.

Third, it is necessary to investigate what facilitates or obstructs an individual’s use of an IPS in a hospital. Two of the most critical success factors in relation to information technology projects in hospitals considered are: (1) the complexity of the system and (2) the explanation of how to access it [45]. For the purposes of this investigation, the capability of an individual to use an IPS is dependent on those success factors, which affect whether the individual perceives that she or he controls the new IPS. The individual has to be able to use the IPS under guidance to confirm these control beliefs [46]. Intention to use the system is positively influenced by a higher perceived behavioral control [20,40]. These considerations lead to the following hypotheses:

- H5: The higher the control beliefs concerning an IPS in a hospital, the more positive the perceived behavioral control of an individual regarding the IPS.
- H6: The higher the perceived behavioral control in terms of an IPS in a hospital, the higher the intention to use the IPS.

The navigational skills of the individuals have to be examined to determine confidence in the use of IPS in a hospital (in terms of perceived behavioral control) [47]. Therefore, navigational skills are used here to validate the connection between spatial abilities and intention to use an IPS in a hospital, as well as the connection between spatial abilities and perceived behavioral control. Yao et al [48] determined that spatial abilities are an important predictor of planning to use a navigational application in outdoor environments. Accordingly, we assume that individuals who are good at navigating through buildings without assistance will be confident about using an IPS in a hospital but will not need to use an IPS urgently. Therefore, we differentiate between hospital visitors and hospital employees. For visitors, we investigate their spatial abilities as a whole, formulating the following hypotheses:

- H7: The higher the spatial abilities, the higher the perceived behavioral control.
- H8: The higher the spatial abilities, the lower the intention to use an IPS in a hospital.

For employees, we investigate their spatial abilities both for buildings that they know (the hospital where they work) and for large unfamiliar buildings, leading to the following hypotheses:

- H9: The higher the spatial abilities for known buildings, the higher the perceived behavioral control.
- H10: The higher the spatial abilities for large unknown buildings, the higher the perceived behavioral control.
- H11: The higher the spatial abilities for known buildings, the lower the intention to use an IPS in a hospital.
- H12: The higher the spatial abilities for unknown buildings, the lower the intention to use an IPS in a hospital.

**Figure 3:**
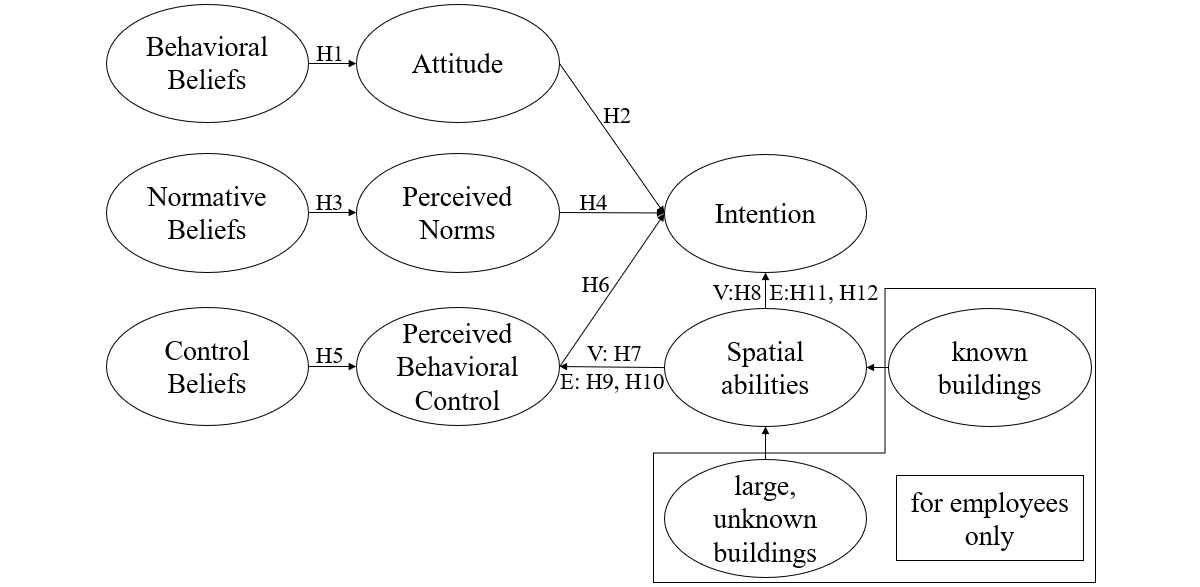
Research model.

The research model developed from these hypotheses is shown in Figure 3.

## Methods

### Measures

We used a 7-point Likert scale for each item (from 1 “do not agree at all” to 7 “completely agree”). As Fishbein and Ajzen [20] noted, “it is important to realize that there is no single reasoned action questionnaire. Each investigation requires the construction of a suitable questionnaire.” We, therefore, adjusted the original framework for RAA research to suit the context of IPS in hospitals, creating items that cover relevant behavioral beliefs about time savings and hygiene considerations. In terms of normative beliefs, it is necessary to differentiate between hospital visitors and hospital employees. For visitors, the most relevant normative reference groups are derived from private life, namely family and close friends. For employees, colleagues in the same functional area, colleagues in related functional areas, and superiors are defined as relevant normative groups. In terms of control beliefs, we include ease of access and the necessity of explaining how the IPS functions.

We included several control variables (ie, the size of the hospital and types of buildings, the employees’ work area, how long employees have been working at the hospital, when visitors or patients were present in the hospital, levels of personal innovativeness, and demographic data such as age and gender). The complete questionnaires can be found in A-1 Multimedia Appendix 1.

### Participants and Data Collection

The crowdworking platform Clickworker (similar to Amazon MTurk) was used to gather hospital visitors and employees in Germany in April and August 2020. The questionnaires for visitors and employees were separate. We included test questions at the beginning and end of the questionnaire to ensure that the self-reported status was correct. At the beginning of the process, participants also received a text that explained the main function of an IPS. Since the unsupervised online platform paid the participants for their responses, we followed the recommendations of Goodman et al [49] by keeping the questionnaire short and enriching it with attention checks.

Among the hospital visitors, the youngest participant was 18 years of age, and the oldest was 68 years. The mean age was 36.08 years (SD 11.73), with a variance of 137.48 years. A majority (250/323, 77.4%) were aged between 18 and 44 years, and 22.29% (72/323) were between 45 and 64 years.

For the hospital employees, the mean age was 33.67 years (SD 9.62), with a variance of 92.37 years. We asked the employees to state the main functional area in which they work. The most common area was nursing care (96/304, 31.58%), followed by hospital management (51/304, 16.78%), building services (37/304, 12.17%), diagnosis and therapy (26/304, 8.55%), research, teaching, and training (20/304, 6.58%), emergency medical services (19/304, 6.25%), pastoral care and social services (16/304, 5.26%), supply and waste management (12/304, 3.95%), integrated ambulant care (12/304, 3.95%), kindergarten for employees (11/304, 3.62%), hospice care (3/304, 0.97%), and patient accommodation (1/304, 0.33%).

### Validity and Reliability

A partial least squares approach to SEM was used to test the proposed models for hospital visitors and employees. Variance-based SEM is more suitable than covariance-based SEM in cases where the aim is to explain and predict the target construction in structural models or to identify key drivers [50]. Multiple regression analysis, an example of variance-based SEM, develops parameters that “maximize the explained variance of dependent constructs” [50]. We used SmartPLS (version 3.3.3; SmartPLS GmbH) to evaluate our models, estimating our weightings with a path method and determining the significance of the path coefficients using bootstrapping procedures with 5000 samples [50]. We followed the requirements of Hair et al [50] and Hulland [51] by testing (1) internal consistency reliability, (2) indicator reliability, (3) convergent validity, and (4) discriminant validity.

First, composite reliability, used to examine internal consistency, was confirmed for both visitors and employees (A-5 Multimedia Appendix 1). Second, we investigated the reliability of the indicators concerning the reflective variables “attitude,” “perceived norms,” and “perceived behavioral control” and found the requirements to be fulfilled for both groups (A-2 Multimedia Appendix 1). Third, convergent validity in terms of the reflective variables was confirmed for both groups (A-5 Multimedia Appendix 1). Fourth, the discriminant validity of our measures was investigated using heterotrait-monotrait ratios and confirmed for both groups (A-6 Multimedia Appendix 1). Thus, we conclude that the reliability and validity of the reflective measures are adequate.

The variance inflation factor was used to check for multicollinearity among the indicators for formative belief variables. For both groups, the values were in line with requirements (A-3 Multimedia Appendix 1). The outer weights and loadings used to test the relative and absolute importance of indicators were all significant for both groups (A-4 Multimedia Appendix 1). To check heterogeneity between the indicators, we determined whether the bivariate correlations were higher between an indicator and the variable than between the indicators [52]. Investigation of the results identified no suppressors and no collinear indicators for either group.

We also conducted several tests to ascertain the quality of our structural model. We used the standardized root mean square residual (SRMR) to determine the approximate fit for our composite factor and common factor models [53]. We obtained .075 for the SRMR composite factor model for the visitors and .10 for the SRMR common factor model. For the employees, the values were .55 and .085, respectively. To evaluate the prediction relevance of the models [54], we followed the literature in using blindfolding procedures with an omission distance of 7 [55]. Both tests yielded positive Stone–Geisser Q2 values (A-7 Multimedia Appendix 1), allowing us to conclude that the models have strong overall predictive power [54].

## Results

The descriptive statistics and correlations for both our samples are given in Table 1. Note that variable 7 applies to visitors and variables 8 and 9 to employees only.

**Figure 4:**
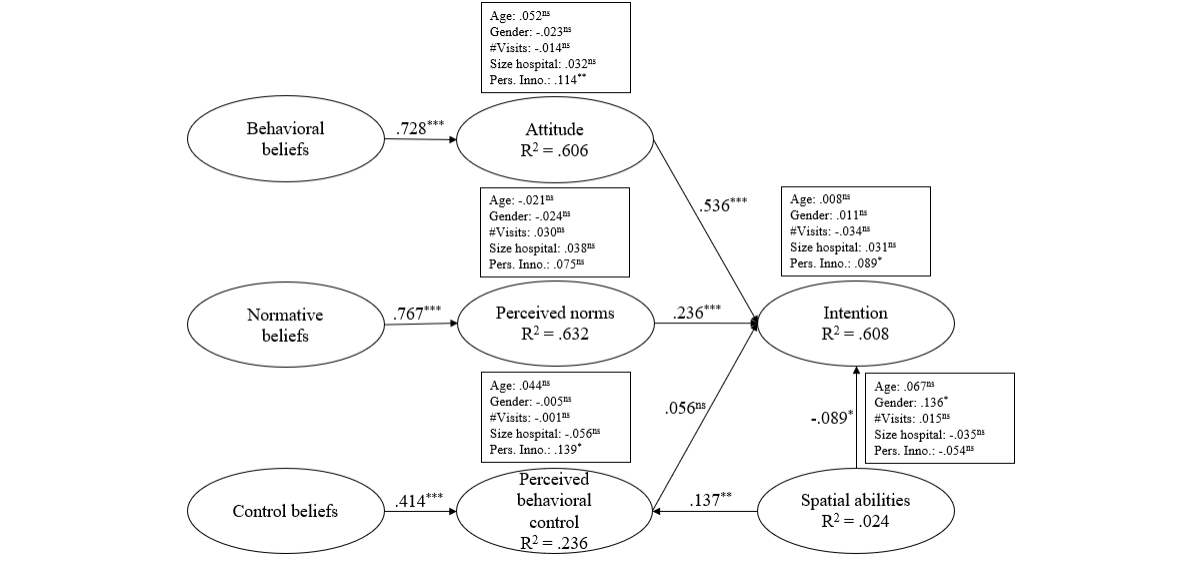
Research model results for hospital visitors. *P<.05; **P<.01; ***P<.001; ns: not significant.

The results of our analysis concerning the hospital visitors are presented in Figure 4.

For the visitors, strong empirical evidence was found in support of H1 (*β*=.728; *P*<.001; *f²*=1.153), H3 (*β*=.767; *P*<.001; *f²*=1.389), and H5 (*β*=.414; *P*<.001; f²=0.179), which indicates that the respective beliefs are relevant antecedents. Furthermore, an increase in R^2^ concerning behavioral beliefs resulted in a higher positive attitude, and 60.6% of the variance can be explained by the behavioral beliefs. Regarding the normative beliefs, the explainable variance in perceived norms is similarly strong (63.2%). In contrast, the variance explained by the control beliefs toward perceived behavioral control is comparatively low (23.6%).

Our investigation of H2 (*β*=.536; *P*<.001; *f²*=.381), H4 (*β*=.236; *P*<.001; *f²*=.087), and H8 (*β*=–.089; *P*=.015; *f²*=.019) supported H2 and H4 but not H8. We determined that attitude has a strong influence on intention to use an IPS in a hospital and that perceived norms (as assessments of the intentions of family and close friends) also have an influence. When we consider navigational skills, it is conspicuous that H8 yields a negative value, suggesting that an increase in spatial abilities leads to a lower intention to use IPS in a hospital.

**Figure 5:**
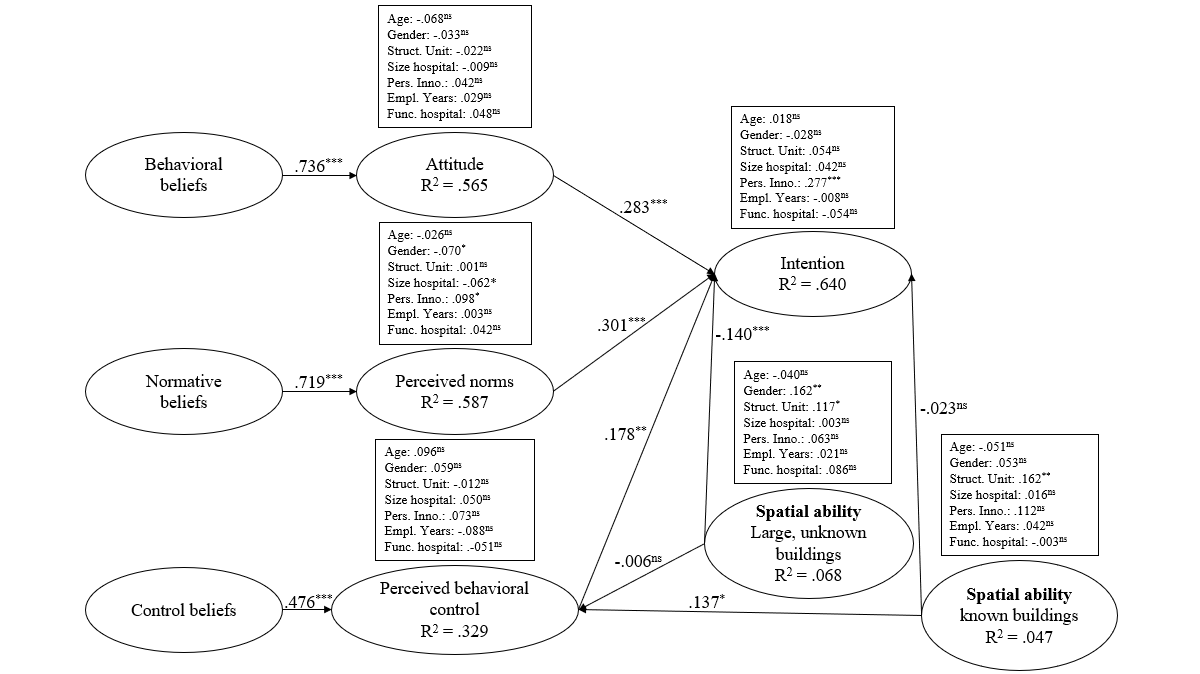
Research model results for hospital employees. *P<.05; **P<.01; ***P<.001; ns: not significant.

We found that perceived behavioral control is not a predictor of intention to use an IPS (*β*=.056; *P*=.129; *f²*=.006). H6 is therefore not supported. In contrast, H7 is supported, as spatial abilities are a predictor of perceived behavioral control (*β*=.137; *P*<.001; *f²*=.023). We used control variables to verify the research model further and found that they had no significant influence, with the exception of personal innovativeness on attitude (*β*=.114; *P*=.001; *f²*=0.029) and on perceived behavioral control (*β*=.139; *P*=.013; *f²*=0.020). The results of the research model regarding hospital employees are summarized in Figure 5.

**Table 1:** Descriptive statistics for the overall sample and correlations among variables for visitors (V) and employees (E).

Variable^a^	Mean (SD)	1	2	3	4	5	6	7	8	9	10
1	V^b^: 33.04 (11.2) E: 29.94 (11.63)	–^c^	V: .51^***^ E: .70^**^	V: .67^***^ E: .71^**^	V: .77^***^ E: .74^**^	V: .53^***^ E: .63^**^	V: .38^***^ E: .51^**^	V: –.21^***^	E: –.04	E: .20^**^	V: .70^***^ E: .70^**^
2	V: 23.75 (10.44) E: 27.35 (10.34)		–	V: .52^***^ E: .688^**^	V: .56^***^ E: .63^**^	V: .79^***^ E: .74^**^	V: .21^***^ E: .54^**^	V: .03	E: .01	E: .21^**^	V: .49^***^ E: .62^**^
3	V: 30.24 (11.27) E: 29.92 (11.84)			–	V: .67^***^ E: .67^**^	V: .58^***^ E: .65^**^	V: .28^***^ E: .50^**^	V: –.15^**^	E: –.08	E: .19^**^	V: .71^***^ E: .70^**^
4	V: 5.55 (1.13) E : 5.39 (1.14)				–	V: .59^***^ E: .66^**^	V: .36^***^ E: .43^**^	V: –.16^**^	E: .05	E: .11^**^	V: .74^***^ E: .65^**^
5	V: 4.64 (1.30) E: 4.84 (1.23)					–	V: .15^**^ E: .39^**^	V: –.09	E: .09	E: .07	V: .59^***^ E: .66^**^
6	V: 6.04 (0.94)E: 5.39 (1.24)						–	V: .10	E: .03	E: .24^**^	V: .31^***^ E: .48^**^
7	V: 4.26 (1.25)							–	–	–	V: –.19^**^
8	E: 4.05 (1.50)								–	E: .43^**^	E: –.10
9	E: 5.30 (1.08)									–	E: .04
10	V: 5.34 (1.54) E: 5.24 (1.47)										–

^a^Number assignment: 1=behavioral beliefs; 2=normative beliefs; 3=control beliefs; 4=attitude; 5=perceived norms; 6=perceived behavioral control; 7=spatial ability; 8=spatial ability large, unknown buildings; 9=spatial ability known buildings; 10=intention.

^b^V: n=323; E: n=304.

^c^Not applicable.

*P<.05; **P<.01; ***P<.001; one-tailed tests.

We found strong empirical evidence for H1 (*β*=.736; *P*<.001; *f²*=1.038), H3 (*β*=.719; *P*<.001; *f²*=0.999), and H5 (*β*=.476; *P*<.001; *f²*=0.244), which again indicates that these beliefs are relevant antecedents. The R² results were similar to those for the visitors’ model, in that the behavioral and normative beliefs have a strong influence on attitude (56.5%) and perceived norms (58.7%). The influence of the control beliefs on perceived behavioral control (32.9%) is higher than in the visitors’ model.

The results support H2 (*β*=.283; *P*<.001; *f²*=0.114), H4 (*β*=.301; *P*<.001; *f²*=0.126), H6 (*β*=.178; *P*<.001; *f²*=0.062), H11 (*β*=–.023; *P*=.310; *f²*=0.001), and H12 (*β*=–.140; *P*<.001; *f²*=0.041), although the results for H11 are not significant. Thus, all the reflective variables of the RAA (attitude, perceived norms, and perceived behavioral control) are significant for intention to use. Moreover, in line with H12, positive spatial abilities concerning large unknown buildings negatively influence intention to use. Investigation of H9 (*β*=.137; *P*=.014; *f²*=0.001) and H10 (*β*=–.006; *P*=.460; *f²*=0.000) showed that the correlations are not significant. We also established that our control variables, with the exception of gender, had no significant influence on spatial abilities for large unknown buildings (*β*=.162; *P*=.003; *f²*=0.027), personal innovativeness on intention to use (*β*=.277; *P*<.001; *f²*=0.168), and the structural unit in which the employees are employed on spatial abilities for known buildings (*β*=.162; *P*=.006; *f²*=0.023).

As gender and age are important factors in spatial ability [56-61] and personal innovativeness [62,63], we post hoc analyzed our data accordingly. We divided the data set into subgroups for women (visitors: n=109; employees: n=124) and men (visitors: n=211; employees: n=178), for ages 18-33 years (n=162) and 34-68 years (n=161) for visitors (combining both genders), and for ages 18-32 years (n=152) and 33-63 years (n=152) for employees. Table 2 gives the significances for the respective groups.

**Table 2:** Post hoc analysis by gender and age.

No.	Group	Gender	Age, years	Correlation/(*f²*)	*β*	*P* value
1	Visitors	Women	All	SA^a^>PBC^b^/(0.025)	.226	.008
2	Visitors	Men	All	SA>PBC	.061	.188
3	Visitors	Both	34–68	SA>PBC	.216	.001
4	Visitors	Both	18–33	SA>PBC	.058	.235
5	Visitors	Women	34–68	SA>PBC	.300	.018
6	Visitors	Women	18–33	SA>PBC	.172	.098
7	Visitors	Men	34–68	SA>PBC	.133	.078
8	Visitors	Men	18–33	SA>PBC	–.034	.371
9	Employees	Women	All	SA/KB^c^>PBC/(0.020)	.109	.162
10	Employees	Men	All	SA/KB>PBC	.172	.018
11	Employees	Both	33–63	SA/KB>PBC	.071	.232
12	Employees	Both	18–32	SA/KB>PBC	.177	.025
13	Employees	Women	33–63	SA/KB>PBC	.245	.042
14	Employees	Women	18–32	SA/KB>PBC	.353	.002
15	Employees	Men	33–63	SA/KB>PBC	.169	.046
16	Employees	Men	18–32	SA/KB>PBC	.141	.138
17	Employees	Women	All	SA/LUB^d^>I^e^/(0.041)	–.154	.008
18	Employees	Men	All	SA/LUB>I	–.104	.026
19	Employees	Both	33–63	SA/LUB>I	–.212	.000
20	Employees	Both	18–32	SA/LUB>I	–.076	.086
21	Employees	Women	33–63	SA/LUB>I	–.078	.161
22	Employees	Women	18–32	SA/LUB>I	–.044	.299
23	Employees	Men	33–63	SA/LUB>I	–.161	.019
24	Employees	Men	18–32	SA/LUB>I	–.009	.453
25	Visitors	Women	All	PI^f^>Att^g^/(0.024)	.094	.072
26	Visitors	Men	All	PI>Att	.113	.011
27	Visitors	Both	34–68	PI>Att	.027	.285
28	Visitors	Both	18–33	PI>Att	.200	.000
29	Visitors	Women	34–68	PI>Att	–.060	.239
30	Visitors	Women	18–33	PI>Att	.223	.006
31	Visitors	Men	34–68	PI>Att	.087	.103
32	Visitors	Men	18–33	PI>Att	.119	.043
33	Visitors	Women	All	PI>PBC	.189	.047
34	Visitors	Men	All	PI>PBC	.119	.045
35	Visitors	Both	34–68	PI>PBC	.175	.018
36	Visitors	Both	18–33	PI>PBC	.100	.138
37	Visitors	Women	34–68	PI>PBC	.237	.090
38	Visitors	Women	18–33	PI>PBC	.181	.153
39	Visitors	Men	34–68	PI>PBC	.163	.052
40	Visitors	Men	18–33	PI>PBC	.047	.337
41	Employees	Women	All	PI>I/(0.168)	.248	.001
42	Employees	Men	All	PI>I	.319	.000
43	Employees	Both	33–63	PI>I	.243	.000
44	Employees	Both	18–32	PI>I	.315	.000
45	Employees	Women	33–63	PI>I	.186	.040
46	Employees	Women	18–32	PI>I	.201	.039
47	Employees	Men	33–63	PI>I	.238	.003
48	Employees	Men	18–32	PI>I	.440	.000

^a^SA: spatial ability.

^b^PBC: perceived behavioral control.

^c^KB: known buildings.

^d^LUB: large unknown buildings.

^e^I: intention.

^f^PI: personal innovativeness.

^g^Att: attitude.

## Discussion

### Principal Findings

In our investigation of intention to use an IPS in a hospital, we identified significant differences between visitors and employees. First, while perceived behavioral control is not significant in determining visitors’ intention to use (*β*=–.056; *P*=.129; *f²*=0.016), it is significant for employees (*β*=.178; *P*=.005; *f²*=0.062). Thus, active control over the intention to use an IPS is more relevant for employees than visitors. This might reflect the fact that employees are more experienced than visitors in finding their way around a hospital. Other studies concerning navigational [48] and health care–related [64,65] occupational contexts seem to support this theory.

Second, spatial abilities are significant for perceived behavioral control regarding hospital visitors (*β*=.137; *P*=.006; *f²*=0.023) and known buildings (*β*=.137; *P*=.014; *f²*=0.020). However, they are not significant for large unknown buildings from the viewpoint of hospital employees (*β*=–.006; *P*=.460; *f²*=0.000). Thus, the urgency of using an IPS in a building known to the employee (eg, the hospital where she or he is employed) is lower if the employee’s spatial abilities are high, but this is not the case for large unknown buildings. Likewise, spatial abilities are not a predictor of visitors’ intention to use an IPS (*β*=–.089; *P*=.015; *f²*=0.016) or the spatial abilities of employees with regards to known buildings (*β*=–.023; *P*=.310; *f²*=0.001). In contrast, spatial abilities are a predictor for employees using an IPS with respect to large unknown buildings (*β*=–.140; *P*<.001; *f²*=0.041), which indicates that employees have an intention to use an IPS if the building is large and unfamiliar.

For visitors, personal innovativeness is not significant for intention to use an IPS (*β*=.089; *P*=.022; *f²*=0.016); however, it is significant for employees (*β*=.277; *P*<.001; *f²*=0.168). This insight aligns with previous research, as personal innovativeness is an important predictor of behavioral intention [56,57].

In current research on spatial abilities, the influence of gender is disputed; research that uses abstract measures, such as mental rotation, indicates that men are better than women at wayfinding [58,59], while research in indoor contexts has identified no major gender differences [64,65]). In this study, for hospital visitors, we found that the older age group (those aged 34-68 years), and especially women, tend to be more realistic about their spatial abilities and their need to use an IPS (see Table 2, numbers 1-8). This suggests that women have greater feelings of uncertainty about wayfinding in a building. However, although women are more likely to use navigation systems [48], actual wayfinding performance does not differ by gender [66].

The findings concerning the impact of the spatial abilities of employees for known buildings on perceived behavioral control align with the findings for visitors. However, it should be noted that the path is also significant for male employees aged 33-63 years (see Table 2, numbers 9-16). These results support the view that physical age and improved experience are positively related, as navigational experience initially increases with age [48], before decreasing in elderly people (an age group not represented in this study) [60,61]. The results in relation to large unknown buildings show that, for both genders and all the age groups under study, higher spatial abilities lead to lower intention to use an IPS in a hospital. However, there is some discrepancy in the results for the different age groups, with a significance for men aged 33-63 years (see Table 2, numbers 17-24), which we ascribe to experience in navigation [48].

For unfamiliar environments, other aspects may be more relevant in determining the urgency of navigational assistance and thus intention to use an IPS, such as the complexity of the environment [67]. In terms of the influence of personal innovativeness on the attitude of hospital visitors, we determined that the path is significant for men and for younger individuals (those aged 18-33 years; see Table 2, numbers 25-32). Concerning personal innovativeness and perceived behavioral control, the path is mainly driven by older participants and is independent of gender (see Table 2, numbers 33-40).

Hence, our results support the consensus in technology adoption research that there is a gender difference. Men’s decisions to adopt new technology are driven mainly by their attitude toward the technology, whereas women’s decisions are driven by subjective norms and perceived behavioral control [63]. Concerning the influence of personal innovativeness on attitude and perceived behavioral control, research has determined that attitude toward new technology is more relevant for younger workers, whereas perceived norms and perceived behavioral control are more relevant for older workers [62]. Our findings support these insights by identifying a positive influence of personal innovativeness on intention to use for all genders and age groups (see Table 2, numbers 41-48). An IPS is aimed at individuals who like to explore and experiment with new information technologies, which is a common perception in research on information system adoption and use [68,69].

To clarify the influence of the employees’ structural unit on their spatial abilities for known buildings, we post hoc analyzed our data set according to the functional areas in which the individuals are employed. Thus, we distinguished between employees who move through hospital buildings frequently because of their occupation (ie, those in nursing care, building services, and emergency medical services) and those who work mainly in the same place (all the other functional areas represented in our data; see “Data Collection And Participants”). We found that employees who work mainly in the same place are more confident in their spatial abilities in relation to known buildings (*β*=.194; *P*=.017, *f²*=0.023) than those participants frequently moving (*β*=.088; *P*=.172), which we ascribe to the fact that those employees who work mainly in the same place have a lower range of motion in the hospital and have to know a fewer number of floors or buildings, respectively.

Concerning the core model of the RAA, our investigation indicates that attitude and perceived norms are strong predictors of intention to use an IPS in a hospital. For hospital employees, the results are more differentiated; all the reflective variables of the RAA (attitude, perceived norms, and perceived behavioral control) are significant for intention to use, with perceived norms having the strongest influence. Attitude driven by behavioral beliefs is a major predictor of intention to use [20]. Our model indicates that this is the case for hospital visitors and confirms that it is important for hospital employees. In terms of perceived norms, rational choice theorists argue that individual behavior is usually conducted in accordance with self-interest and that we, therefore, accept social norms as limits on those behaviors. In this article, we ensure that social norms do not represent an individual’s interest only but that of a larger social system [70]. We established that perceived norms significantly influence intention to use the system for both hospital visitors (with family and close friends as the reference groups) and hospital employees. Moreover, perceived norms are the most important predictors for employees, reflecting the importance of recommendations from immediate colleagues and colleagues working in other functional areas and superiors.

The descriptive statistics for spatial abilities show a mean of 4.18 (SD 1.56) for visitors, and for employers, a mean of 4.05 (SD 1.69) for large unknown buildings and a mean of 5.29 (SD 1.34) for known buildings. These results indicate that employees tend to navigate better through known buildings than through large unknown buildings, although no such tendency is found for visitors. For the influence of the personal innovativeness of employees on their intention to use an IPS, the mean value of 4.86 (SD 1.54) suggests that employees intend to use an IPS if they are personally innovative in terms of new technologies (see A-1 Multimedia Appendix 1). The mean values for all three intention items (on a scale of 1 to 7; visitors: n=323 and employees: n=304) are as follows: intention 1 (visitors: mean 5.35, SD 1.60; employees: mean 5.36, SD 1.53); intention 2 (visitors: mean 5.39, SD 1.57; employees: mean 5.23, SD 1.58); intention 3 (visitors: mean 5.27, SD 1.61; employees: mean 5.15, SD 1.57). Thus, our model indicates that both visitors and employees have a positive intention to use an IPS. Accordingly, we recommend that hospitals pursue IPS implementation.

### Conclusions

We analyzed the relevance of IPS in hospitals by considering the perspectives of the main actors, visitors, and employees. The explained variance indicates that intention to use is well predicted and that relevant aspects in the context are covered. This confirms that RAA is an appropriate approach for determining intention to use an IPS in a hospital. Furthermore, our results show that individual attitude and the social norms of relevant reference groups positively impact intention to use an IPS in a hospital. For employees, perceived behavioral control also positively influences intention to use an IPS. These results have many implications for theory, practice, and future research.

### Theoretical Implications

Our study design and findings contribute to the literature in several ways. First, we add to the knowledge of how systems or applications, specifically IPS, in the health care management context are accepted by actors in a hospital. Whereas related work regarding general health care tracking apps, including COVID-19-related apps [14], has focused on general use with a broad public interest, we provide insights into a spatially limited organizational context.

Second, we integrate two major stakeholder groups into our analysis: general users, such as patients or visitors, and professional staff. As such, we demonstrate how health care management applications are perceived from a nonexpert perspective, thereby building on previous research, which has generally adopted an expert perspective [15].

Third, we introduce the RAA to analyze intention to use applications in the health care management context, thereby extending the theory conceptually and empirically into a context that considers spatial abilities and personal innovativeness. The high explained variance confirms that the theory is helpful for understanding the reasons for adoption intentions. This increased focus on analyzing the influence of different beliefs from a functional perspective extends other theories that have been applied in the context, such as uncertainty reduction theory [14] and protection motivation theory [71].

Fourth, our extension of the RAA to cover spatial abilities and personal innovativeness contributes to the understanding of gender-related and age-related spatial ability. Hence, we demonstrate that demographics matter and should be considered when analyzing the acceptance of applications in a health care management context.

### Practical Implications

From a practical perspective, we recommend that hospitals invest in implementing IPS, as our results show that the potential user intention is high. Furthermore, IPS market research forecasts indicate that low-energy Bluetooth will be one of the most lucrative segments of the IPS market [10], thanks to the low hardware costs and low energy consumption [72]. These forecasts lend support to our recommendation.

However, the IPS design requirements of hospital visitors and employees are different. From our finding that visitors’ attitudes and perceived norms are the most important predictors of their intention to use, it follows that the system needs to be simple and self-explanatory. The main focus of the application should be navigation to specific rooms or points of interest. If those services function properly, visitors are likely to recommend the system to reference groups that are important to them (eg, close friends and family), who will then assess and use the system accordingly.

For hospital employees, attitude and perceived norms are also relevant. However, the system needs a different functional focus for employees, whose intentions are determined by perceived behavioral control. Our research model shows that employees that work mainly in the same place are confident in their spatial abilities for known buildings. In other words, they do not need navigational services for specific rooms or points of interest in the hospital building in which they are employed. Asset tracking, in contrast, is more relevant, as this can facilitate daily work and help reduce redundant activity.

### Limitations and Future Research

Our study is subject to some limitations that inform future research. First, we used the crowdworking platform Clickworker to gather our participants. This decision partly predetermined the personal innovativeness of our respondents, as individuals who use digital platforms are likely to be more personally innovative than those who respond to a pen and paper survey. Second, our study design involves convenience sampling, albeit with specific criteria for participation. Thus, we cannot claim that our sample is representative, and further research should focus on a defined target population. Third, our participants are from a single country, Germany. Future studies should cover different countries to identify additional relevant factors. Fourth, our research does not consider other settings, such as large hardware stores, that may be relevant to and interact with the hospital context. Therefore, future research should investigate general acceptance of IPS by, for example, determining the likelihood of using an IPS in a hardware store after using it in a hospital.

## Appendix

Multimedia Appendix 1 The Multimedia Appendix contains: the questionnaire (A-1), loadings of reflective variables (A-2), VIF values (A-3), loadings and weights of formative variables (A-4), composite reliability and AVE (A-5), HTMT- (A-6) and Stone-Geisser-values (A-7) of our study.

## Abbreviations

IPS: indoor positioning system
RAA: reasoned action approach
SEM: structural equation modeling
SRMR: standardized root mean square residual
