# Supplementary material for: Factors Influencing the Intention of Actors in Hospitals to Use Indoor Positioning Systems: Reasoned Action Approach
Source: J Med Internet Res. 2021 Oct 5;23(10):e28193. doi: 10.2196/28193 (PMC8527384; doi:10.2196/28193)
Supplement: Multimedia Appendix 1 [file jmir_v23i10e28193_app1.docx]

# Multimedia Appendix

## A-1: Questionnaire

As two different surveys were conducted, two different questionnaires exist. Therefore, the statements following “B” belong to both questionnaires, whereas arguments containing “V” represent hospital visitors and “E” representing the hospital employees.

| B: .5 – control question – size of the hospital |
| --- |
| B: Which building structure has the hospital that you have visited most frequently in the past 365 days? |
| B: -1- hospital with the structure of an office building |
| B: -2- hospital with the structure of a skyscraper |
| B: -3- hospital that is distributed over several buildings |
| B: -4- hospital that is distributed over a large area |
| E: 0.75 – control question – functional area in the hospital |
| E: Which of the following functional areas is the one you are mostly working in? |
| E: -1- Diagnosis and Therapy |
| E: -2- Nursing Care |
| E: -3- Nursing Home Management |
| E: -4- Hospital Management |
| E: -5- Supply- and Waste Management |
| E: -6- Research, Teaching and Training |
| E: -7- Pastoral Care and Social Services |
| E: -8- Emergency Medical Service |
| E: -9- Kindergarten for Employees |
| E: -10- Patient Hotel |
| E: -11- Medical Advisory Service of the Statutory Health Insurance Funds |
| E: -12- Hospice |
| E: -13- Integrated Ambulant Care |
| E: -14- Building Services |
| B: 1 – behavioral beliefs |
| E: -1- An application for indoor navigation would help me to get the information needed to find devices that I need to conduct my work. |
| E: -2- It is important to me to get the information needed to find devices that I need to conduct my work. |
| B: -(V:1)(E:3)- An application for indoor navigation would help me move hygienically along the shortest routes in the hospital. |
| B: -(V:2)(E:4)- It is important to me to move as hygienic as possible through the hospital. |
| B: -(V:3)(E:5)- An application for indoor navigation would be an appropriate solution to help me find my destination. |
| B: -(V:4)(E:6)- The ease of use of applications for indoor navigation is very important to me. |
| B: 2 – attitude |
| B: The use of an application for indoor navigation would be… |
| B: -1- … advantageous. |
| B: -2- … satisfactory. |
| B: -3- … important. |
| B: -4- … enjoyable. |
| B: -5- I would like the use of an application for indoor navigation. |
| B: 3 – normative beliefs |
| V: -1- My family would advise me to use applications for indoor navigation. |
| V: -2- I generally take my family’s advice very seriously. |
| V: -3- My best friends would advise me to use applications for indoor navigation. |
| V: -4- I generally take my best friends’ advice very seriously. |
| E: -1- My colleagues in other functional areas in the hospital would advise me to use applications for indoor navigation. |
| E: -2- I generally take some advice from colleagues in other functional areas in the hospital very seriously. |
| E: -3- My colleagues in the same functional area in the hospital would advise me to use applications for indoor navigation. |
| E: -4- I generally take some advice from colleagues in the same functional area in the hospital very seriously. |
| E: -5- My superior would advise me to use applications for indoor navigation. |
| E: -6- I generally take some advice from my superior very seriously. |
| B: 4 – perceived norms |
| B: -1- Individuals from whom I let myself be influenced would advise me to use applications for indoor navigation. |
| B: -2- Individuals who are important to me would advise me to use applications for indoor navigation. |
| B: -3- Individuals whose opinion I appreciate would advise me to use applications for indoor navigation. |
| B: -4- Individuals in a situation comparable to myself would advise me to use applications for indoor navigation. |
| B: 5 – control beliefs |
| B: -1- I would use this application for indoor navigation because it would be easily accessible to me. |
| B: -2- The easy accessibility to applications for indoor navigation is very important to me. |
| B: -3- I would use this application for indoor navigation as far as I get the application explained accordingly. |
| B: -4- The explanation of the use of an application for indoor navigation is very important to me. |
| B: 6 – perceived behavioral control |
| B: -1- It is under my control to use applications for indoor navigation. |
| B: -2- It is mainly up to me to use applications for indoor navigation. |
| B: -3- I am convinced that I can use applications for indoor navigation. |
| B: -4- If I really want to, I can use applications for indoor navigation. |
| B: 7 – intention |
| B: -1- I would definitely use such an application for indoor navigation during my next visit to a hospital if it would be available. Mean Value: V: 5.35; E: 5.36, Standard Deviation: V: 1.60; E: 1.53 |
| B: -2- I intend to use such an application for indoor navigation during my next visit to a hospital if it would be available. Mean Value: V: 5.39; E: 5.23, Standard Deviation: V: 1.57; E: 1.58 |
| B: -3- I plan to use such an application for indoor navigation during my next visit to a hospital if it would be available. Mean Value: V: 5.27; E: 5.15, Standard Deviation: V: 1.61; E: 1.57 |
| B: 8 – spatial abilities |
| V: -1- I am good in navigating myself through buildings. Mean Value: 4.64, Standard Deviation: 1.51 |
| V: -2- I always find the shortest way through buildings while I am navigating myself. Mean Value: 3.95, Standard Deviation: 1.61 |
| V: -3- I do not need assistance while navigating myself through buildings. Mean Value: 3.95, Standard Deviation: 1.60 |
| E: -1- I am good in navigating myself through buildings that are large and unknown to me. Mean Value: 4.37, Standard Deviation: 1.61 |
| E: -2- I am good in navigating myself through buildings that are known to me. Mean Value: 5.49, Standard Deviation: 1.27 |
| E: -3- I always find the shortest way through buildings that are large and unknown to me, while I am navigating myself. Mean Value: 3.90, Standard Deviation: 1.76 |
| E: -4- I always find the shortest way through buildings that are known to me, while I am navigating myself. Mean Value: 5.19, Standard Deviation: 1.29 |
| E: -5- I do not need assistance while navigating myself through buildings, that are large and unknown to me. Mean Value: 3.89, Standard Deviation: 1.70 |
| E: -6- I do not need assistance while navigating myself trough buildings, that are known to me. Mean Value: 5.21, Standard Deviation: 1.45 |
| B: 9 – personal innovativeness |
| B: -1- When I hear about new information technology, I look forward to experimenting with it. Mean Value: V: 5.36; E: 5.32, Standard Deviation: V: 1.37; E: 1.35 |
| B: -2- I am usually the first of my friends to try new information technology. Mean Value: V: 4.37; E: 4.68, Standard Deviation: V: 1.68; E: 1.61 |
| B: -3- Basically, I am reluctant to try out new information technologies. Mean Value: V: 3.21; E: 4.29, Standard Deviation: V: 1.61; E: 1.76 |
| B: -4- I like to experiment with new information technologies. Mean Value: V: 5.12; E: 5.13, Standard Deviation: V: 1.43; E: 1.45 |

## A-2: Loadings of reflective variables

| Cons-truct | Item | Loadings |
| --- | --- | --- |
| Attitude | The use of an application for indoor navigation would be… | |
|  | -1- … advantageous. | V: 0.908  E: 0.879 |
|  | -2- … satisfactory. | V: 0.852  E: 0.789 |
|  | -3- … important. | V: 0.833  E: 0.774 |
|  | -4- … enjoyable. | V: 0.86  E: 0.876 |
|  | -5- I would like the use of an application for indoor navigation. | V: 0.924  E: 0.896 |
| Perceived Norms | -1- Individuals from whom I let myself be influenced would advise me to use applications for indoor navigation. | V: 0.918  E: 0.874 |
|  | -2- Individuals who are important to me would advise me to use applications for indoor navigation. | V: 0.948  E: 0.908 |
|  | -3- Individuals whose opinion I appreciate would advise me to use applications for indoor navigation. | V: 0.948  E: 0.893 |
|  | -4- Individuals in a situation comparable to myself would advise me to use applications for indoor navigation. | V: 0.863  E: 0.887 |
| Perceived Behavioral Control | -1- It is under my control to use applications for indoor navigation. | V: 0.761  E: 0.807 |
|  | -2- It is mainly up to me to use applications for indoor navigation. | V: 0.825  E: 0.822 |
|  | -3- I am convinced that I can use applications for indoor navigation. | V: 0.849  E: 0.847 |
|  | -4- If I really want to, I can use applications for indoor navigation. | V: 0.853  E: 0.870 |
| Intention | -1- I would definitely use such an application for indoor navigation during my next visit to a hospital if it would be available. | V: 0.954  E: 0.936 |
|  | -2- I intend to use such an application for indoor navigation during my next visit to a hospital if it would be available. | V: 0.975  E: 0.957 |
|  | -3- I plan to use such an application for indoor navigation during my next visit to a hospital if it would be available. | V: 0.973  E: 0.923 |

## A-3: VIF values

| Item | VIF |
| --- | --- |
| B: 1 – behavioral beliefs | |
| E: “-1- An application for indoor navigation would help me to get the information needed to find devices that I need to conduct my work.” X E: “-2- It is important to me to get the information needed to find devices that I need to conduct my work.” | E: 2.011 |
| B: “-(V:1)(E:3)- An application for indoor navigation would help me move hygienically along the shortest routes in the hospital.” X B: “-(V:2)(E:4)- It is important to me to move as hygienic as possible through the hospital.” | V: 2.1  E: 2.114 |
| B: “-(V:3)(E:5)- An application for indoor navigation would be an appropriate solution to help me find my destination.” X B: “-(V:4)(E:6)- The ease of use of applications for indoor navigation is very important to me.” | V: 2.1  E: 2.566 |
| B: 3 – normative beliefs | |
| V: “-1- My family would advise me to use applications for indoor navigation.” X V: “-2- I generally take my family’s advice very seriously.” | V: 1.807 |
| V: “-3- My best friends would advise me to use applications for indoor navigation.” X V: “-4- I generally take my best friends’ advice very seriously.” | V: 1.807 |
| E: “-1- My colleagues in other functional areas in the hospital would advise me to use applications for indoor navigation.” X E: “-2- I generally take some advice from colleagues in other functional areas in the hospital very seriously.” | E: 3.018 |
| E: “-3- My colleagues in the same functional area in the hospital would advise me to use applications for indoor navigation.” X E: “-4- I generally take some advice from colleagues in the same functional area in the hospital very seriously.” | E: 3.046 |
| E: “-5- My superior would advise me to use applications for indoor navigation.” X E: “-6- I generally take some advice from my superior very seriously.” | E: 2.386 |
| B: 5 – control beliefs | |
| B: “-1- I would use this application for indoor navigation because it would be easily accessible to me.” X B: “-2- The easy accessibility to applications for indoor navigation is very important to me.” | V: 1.285  E: 1.682 |
| B: “-3- I would use this application for indoor navigation as far as I get the application explained accordingly.” X B: “-4- The explanation of the use of an application for indoor navigation is very important to me.” | V: 1.285  E: 1.682 |

## A-4: Loadings and weights of formative variables

| Construct | Item | Loadings | Weights |
| --- | --- | --- | --- |
| behavioral beliefs | E: (BB1) | E: 0.850 | E: 0.326 |
|  | V: (BB1) / E: (BB2) | V: 0.938 / E: 0.868 | V: 0.574 / E: 0.351 |
|  | V: (BB2) / E: (BB3) | V: 0.918 / E: 0.925 | V: 0.503 / E: 0.451 |
| normative beliefs | V: (NB1) / E: (NB1) | V: 0.884 / E: 0.905 | V: 0.463 / E: 0.316 |
|  | V: (NB2) / E: (NB2) | V: 0.939 / E: 0.969 | V: 0.629 / E: 0.617 |
|  | E: (NB3) | E: 0.815 | E: 0.142 |
| control beliefs | V: (CB1) / E: (CB1) | V: 0.957 / E: 0.995 | V: 1.111 / E: 0.913 |
|  | V: (CB2) / E: (CB2) | V: 0.196 / E: 0.710 | V: -0.327 / E: 0.129 |

## A-5: Composite reliability and AVE

|  | Composite Reliability | Average Variance Extracted (AVE) |
| --- | --- | --- |
| B: attitude | V: 0.943 / E: 0.925 | V: 0.767 / E: 0.713 |
| B: perceived norms | V: 0.956 / E: 0.939 | V: 0.846 / E: 0.793 |
| V: spatial ability | V: 0.906 | V: 0.764 |
| E: spatial ability  large, unknown buildings | E: 0.916 | E: 0.784 |
| E: spatial ability  known buildings | E: 0.849 | E: 0.655 |
| B: intention | V: 0.978 / E: 0.957 | V: 0.936 / E: 0.882 |
| B: personal innovativeness | V: 0.908 / E: 0.882 | V: 0.714 / E: 0.667 |
| B: perceived behavioral control | V: 0.893 / E: 0.903 | V: 0.677 / E: 0.700 |

## A-6: HTMT values

Note that the variables 2 and 5 apply to visitors and variables 11 to 15 to employees only.

|  | B: 1 | *V: 2* | B: 3 | B: 4 | *V: 5* | B: 6 | B: 7 | B: 8 | B: 9 | B:  10 | E:  11 | E: 12 | E:  13 | E:  14 | E:15 |  |
| --- | --- | --- | --- | --- | --- | --- | --- | --- | --- | --- | --- | --- | --- | --- | --- | --- |
| 1 |  |  |  |  |  |  |  |  |  |  |  |  |  |  |  | 1 |
| *2* | V:  .052 |  |  |  |  |  |  |  |  |  |  |  |  |  |  | *2* |
| 3 | V:  .092  E:  .045 | V:  .031 |  |  |  |  |  |  |  |  |  |  |  |  |  | 3 |
| 4 | V:  .028  E:  .027 | V:  .038 | V:  .633  E:  .726 |  |  |  |  |  |  |  |  |  |  |  |  | 4 |
| 5 | V:  .086 | V:  .009 | V:  .188 | V:  .102 |  |  |  |  |  |  |  |  |  |  |  | 5 |
| 6 | V:  .036  E:  .006 | V:  .004 | V:  .126  E:  .038 | V:  .115  E:  .034 | V:  .121 |  |  |  |  |  |  |  |  |  |  | 6 |
| 7 | V:  .055  E:  .041 | V:  .101 | V:  .098  E:  .023 | V:  .010  E:  .054 | V:  .028 | V:  .120  E:  .017 |  |  |  |  |  |  |  |  |  | 7 |
| 8 | V:  .034  E:  .013 | V:  .012 | V:  .782  E:  .705 | V:  .623  E:  .710 | V:  .213 | V:  .073  E:  .042 | V:  .088  E:  .049 |  |  |  |  |  |  |  |  | 8 |
| 9 | V:  .130  E:  .072 | V:  .020 | V:  .310  E:  .346 | V:  .308  E:  .405 | V:  .74 | V:  .181  E:  .103 | V:  .092  E:  .027 | V:  .352  E:  .559 |  |  |  |  |  |  |  | 9 |
| 10 | V:  .043  E:  .027 | V:  .030 | V:  .414  E:  .498 | V:  .173  E:  .448 | V:  .080 | V:  .028  E:  .103 | V:  .028  E:  .073 | V:  .345  E:  .548 | V:  .332  E:  .311 |  |  |  |  |  |  | 10 |
| 11 | E:  .068 |  | E:  .031 | E:  .018 |  | E:  .101 | E:  .100 | E:  .078 | E:  .025 | E:  .061 |  |  |  |  |  | 11 |
| 12 | E:  .027 |  | E:  .072 | E:  .114 |  | E:  .207 | E:  .027 | E:  .116 | E:  .216 | E:  .108 | E:  .148 |  |  |  |  | 12 |
| 13 | E:  .051 |  | E:  .153 | E:  .176 |  | E:  .115 | E:  .031 | E:  .150 | E:  .168 | E:  .306 | E:  .077 | E:  .539 |  |  |  | 13 |
| 14 | E:  .124 |  | E:  .037 | E:  .040 |  | E:  .117 | E:  .056 | E:  .044 | E:  .042 | E:  .047 | E:  .347 | E:  .174 | E:  .198 |  |  | 14 |
| 15 | E:  .597 |  | E:  .041 | E:  .043 |  | E:  .115 | E:  .052 | E:  .025 | E:  .035 | E:  .035 | E:  .048 | E:  .021 | E:  .022 | E:  .029 |  | 15 |
| Number assignment  1 = age; 2 = number of visits; 3 = attitude; 4 = perceived norms; 5 = spatial ability; 6 = gender; 7 = hospital size; 8 = intention; 9 = personal innovativeness; 10 = perceived behavioral control; 11 = function hospital; 12 = spatial ability large, unknown buildings; 13 = spatial ability known buildings; 14 = structural unit hospital; 15 = years of employment (in the hospital) | | | | | | | | | | | | | | | | |

## A-7: Stone-Geisser-values

|  | Q² (=1-SSE/SSO) |
| --- | --- |
| B: attitude | V: 0.457  E: 0.387 |
| B: perceived norms | V: 0.527  E: 0.453 |
| V: spatial ability | V: 0.012 |
| E: spatial ability large, unknown buildings | E: 0.041 |
| E: spatial ability known buildings | E: 0.014 |
| B: intention | V: 0.560  E: 0.549 |
| B: perceived behavioral control | V: 0.147  E: 0.210 |
